# Efficacy of upfront hepatectomy without neoadjuvant chemotherapy for resectable colorectal liver metastasis

**DOI:** 10.1186/s12957-021-02210-9

**Published:** 2021-04-05

**Authors:** Kosuke Ono, Tomoyuki Abe, Akihiko Oshita, Yusuke Sumi, Takuya Yano, Hiroshi Okuda, Manabu Kurayoshi, Tsuyoshi Kobayashi, Hideki Ohdan, Toshio Noriyuki, Masahiro Nakahara

**Affiliations:** 1 Department of Surgery, Onomichi General Hospital, 1-10-23 Hirahara Onomichi, Hiroshima, 722-8508 Japan; 2 Department of Gastroenterological and Transplant Surgery, Applied Life Sciences, Institute of Biomedical and Health Sciences, Hiroshima University, 1-2-3 Kasumi, Hiroshima, 734-8551 Japan; 3 Department of Surgery, Hiroshima Citizens Hospital, 7-33 Motomachi, Hiroshima, 730-8518 Japan

**Keywords:** Beppu’s nomogram score, Neoadjuvant chemotherapy, Hepatectomy

## Abstract

**Background:**

Hepatectomy for resectable colorectal liver metastasis (CRLM) is recommended. However, the efficacy of upfront hepatectomy without neoadjuvant chemotherapy (NAC) is unclear due to the uncertainty of perioperative systemic chemotherapy. Moreover, it is crucial to predict the prognosis when considering perioperative chemotherapy. This study evaluated the impact of neoadjuvant chemotherapy on the prognosis of patients with resectable CRLM and assessed the usefulness of Beppu’s nomogram for predicting prognosis.

**Methods:**

This retrospective study identified 88 consecutive inpatients who underwent primary hepatic resection for CRLM; 58 received neoadjuvant chemotherapy and 30 underwent upfront surgery. Factors associated with recurrence-free survival were identified via univariate and multivariate analysis. Furthermore, propensity score analysis using inverse probability of treatment weighting (IPTW) was performed.

**Results:**

On univariate analysis, poor recurrence-free survival was associated with multiple tumors, advanced primary tumor stage, vascular invasion by the primary tumor, a Beppu’s nomogram score ≥ 6, and neoadjuvant chemotherapy. On multivariate analysis, a Beppu’s nomogram score ≥ 6 and neoadjuvant chemotherapy were independent risk factors for recurrence. Neoadjuvant chemotherapy recipients had a higher incidence of lymph node metastasis and vascular invasion than non-recipients. Propensity score analysis revealed no significant difference in the recurrence-free survival rate between these groups.

**Conclusions:**

Our results show that upfront hepatectomy without neoadjuvant chemotherapy can be considered for resectable CRLM treatment. Beppu’s nomogram score can be a tool for predicting the prognosis of patients with CRLM.

## Background

Colorectal cancer (CRC) is the third most common cancer, and its incidence is increasing worldwide [1]. Hepatectomy is the gold standard treatment for colorectal liver metastasis (CRLM). Owing to recent advancements in perioperative surgical management, unresectable CRLMs can now be safely resected by staged hepatectomy [2]. However, even if curative resection is performed, the postoperative recurrence rate in the remnant liver is high (approximately 75%) [3] and the 5-year survival rate is dismal (33–61%) [4].

Perioperative chemotherapy is a potential strategy for improving the long-term survival of patients with CRLM. There is evidence supporting the efficacy of postoperative adjuvant chemotherapy [5]. However, the efficacy of neoadjuvant chemotherapy (NAC) for resectable CRLM remains controversial. In the EORTC 40983 clinical trial, patients with resectable CRLM who received perioperative NAC had a better 3-year progression-free survival rate than those who received surgery alone [6]. Following this trial, the European Society for Medical Oncology recommended perioperative adjuvant chemotherapy for CRLM [7]. However, a subsequent study of the patient groups in the EORTC 49083 found no significant effect of NAC on the 3- or 5-year overall survival (OS) rate [8]. In addition, a review of chemotherapy treatments for resectable CRLM found no difference in the OS rate between patients who received and those who did not receive preoperative chemotherapy [9]. Thus, the efficacy of upfront hepatectomy without neoadjuvant chemotherapy (NAC) is unclear.

Accurate prediction of prognosis is crucial when considering perioperative chemotherapy strategies. In this study, we used Beppu’s nomogram as a prognostic tool. This nomogram consists of six preoperative factors, is simple to apply, and has been known to predict disease-free survival (DFS) rates in CRLM patients after radical resection [10]. Although this nomogram is simple and convenient for clinical use, it is unclear whether it is useful in combination with recent advances in chemotherapy because it is a nomogram analyzed based on cases from 2000 to 2004. Higuchi et al. validated this nomogram and demonstrated its efficacy [11]. However, this was confirmed in patients from 2006 to 2011. Therefore, the usefulness of Beppu’s score even in these days when treatments for CRLM such as chemotherapy regimens are evolving remains uncertain.

This study examined the efficacy of NAC for CRLM with radical resection and the usefulness of Beppu’s nomogram in predicting prognosis.

## Methods

### Patients

We conducted a retrospective cohort study of patients who underwent primary hepatic resection for CRLM in the Department of Surgery at Onomichi General Hospital between June 2006 and April 2019. It was conducted in accordance with the ethical standards of the 1964 Declaration of Helsinki. The study design was approved by our institutional review board (OJH-201509), and all patients provided informed consent for their treatment.

### Surgery

The initial surgery was performed using a laparoscopic or open method. The extent of resection was determined by the location of the tumor. Partial resection was selected if possible; if not, segment resection or lobectomy was selected to preserve liver function. The Pringle method was used as much as possible to control bleeding. One surgical hepatobiliary team performed the surgery in this study.

### NAC

The standard treatment at our facility has been NAC. The administration period depends on the case and regimen; however, the treatment usually includes six courses. The regimens were as follows: 5-fluorouracil (5FU), leucovorin (LV), and oxaliplatin (FOLFOX) or capecitabine and oxaliplatin (CAPOX); 5FU, LV, and irinotecan (FOLFIRI); and tegafur/gimeracil/oteracil (TS-1) or tegafur/uracil with either LV (UFT/UZEL) or capecitabine. In addition, the FOLFOX and FOLFIRI regimens included a molecularly targeted agent (bevacizumab, cetuximab, or panitumumab) if needed. Upfront hepatectomy was performed in patients who refused to undergo NAC and in those whose general condition contraindicated NAC.

### Morbidity

Survival values were calculated from the date of surgery.

### Follow-up strategy

All patients were followed until death and underwent annual follow-ups consisting of abdominal ultrasonography and laboratory tests for tumor markers, namely, carbohydrate antigen 19-9 (CA19-9) and carcinoembryonic antigen. Dynamic computed tomography (CT) was conducted every 6 months. If a definitive diagnosis of recurrence could not be established based on tumor marker data, ultrasonography-guided biopsy imaging (CT, magnetic resonance imaging, endoscopic ultrasonography, or fluorodeoxyglucose-positron emission tomography) was performed.

### Beppu’s nomogram score

The following six preoperative factors were used to create the nomogram for DFS: synchronous metastasis (3 points); positive primary lymph node (3 points); tumor number, (4 points for 2–4 tumors and 9 points for ≥ 5 tumors); largest tumor diameter > 5 cm (2 points); extrahepatic metastasis at hepatectomy (4 points); and preoperative CA19-9 level > 100 (4 points). Zero, 5, 10, and > 10 points corresponded to estimated median DFS times of > 8.4 years, 1.9 years, 1 year, and < 0.6 years, respectively. The total preoperative Beppu’s nomogram scores ranged from 0 to 25 points.

### Liver metastasis classification and prognostic grade classification

The Japanese Classification of Colorectal Carcinoma H-classifications is based on the number and maximum size of tumors (General Rules for Clinical and Pathologic Studies on Cancer of the Colon, Rectum and Anus, 7th Japanese edition, 2009; H0, no liver metastasis; H1, number of metastasis < 4 and size of the largest tumor < 5 cm; H2, other than H1 or H3; H3, number of metastasis > 5 and size of largest tumor > 5 cm). Prognosis classification combines the H-classification with the degree of lymph node metastasis of the primary lesion and the presence or absence of distance metastasis: grade A, H1 and N0 or N1 and M0; grade B, H1 and N2 and M0 and H2 and N0 or N1 and M0; and grade C, H1 and N3 and M0, H2 and N2 or N3 and M0, or all M1 cases and all H3 cases.

### Statistical analyses

Recurrence-free survival (RFS) rates were determined using the Kaplan-Meier method and the log-rank test. Multivariate analyses for RFS were performed using Cox’s regression model. Appropriate calibration of the model was indicated by a *P*-value of 0.620 in the Hosmer-Lemeshow test, and good discrimination was indicated by a C-statistic of 0.773 with a 95% confidence interval (CI) of 0.662–0.884 and a *P*-value < 0.001. Propensity score analysis using inverse probability of treatment weighting (IPTW) was performed to overcome bias related to the different distributions of the covariates between NAC recipients and non-recipients. In the weighted variables such as CEA, lymph node metastasis, lymphatic invasion, and Beppu’s score, we used a Cox regression model to regress recurrence-free survival between patients with NAC and patients with upfront surgery and used a robust variance estimator [12]. After IPTW processing, differences in RFS between these groups were tested using Cox regression and multiple logistic regression analyses. Two-tailed *P*-values < 0.05 were considered statistically significant, and all analyses were performed using SPSS software (version 24; IBM, Armonk, NY, USA).

## Results

### Patients

In total, 88 patients underwent initial hepatectomy for CRLM at our center.

The patients’ clinicopathological characteristics are outlined in Table 1. Of the 88 patients, 63 (72%) were men and 25 (28%) were women, and the median age was 70 years (Table 1). The primary tumor was located in the colon in 47 (53%) patients and the rectum in 41 (47%) patients; it was left-sided in 67 (76%) patients and right-sided in 12 (14%) patients. Synchronous liver metastasis, metachronous CRLM, and synchronous lung metastasis were detected in 42 (48%), 46 (52%), and 5 (6%) patients, respectively. Moreover, 18 patients (39%) with metachronous liver metastasis received chemotherapy along with colorectal resection for liver metastasis. Forty-six (52%) patients had 1 liver metastasis, 28 (32%) had 2–4 liver metastases, and 14 (16%) had ≥ 5 liver metastases. The median tumor number was 1 (range, 1–15), and the median tumor size was 17 mm. Forty-two (48%) patients were with TNM classification stage I–III and 46 (52%) with stage IV.

**Table 1:** Results of univariate and multivariate analyses of the clinicopathological factors for recurrence-free survival rates

Factors	Univariate analysis		Multivariate analysis
	*N*	3 years (%)	*P*-value
Gender			
Male	63	32.6	-
Female	25	40.9	0.361
BMI			
< 23	39	35.9	-
≥ 23	49	34.4	0.910
Location			
Colon	47	34.8	-
Rectum	41	34.6	0.853
Location			
Right side	12	15.0	
Left side	67	37.2	
Transverse	9	44.4	0.125
Timing of liver metastasis			
Metachronous	46	39.4	
Synchronous	42	30.7	0.190
Synchronous lung metastasis			
Absent	83	35.0	
Present	5	40.0	0.590
**Number of tumors**			
1	46	49.0	
2–4	28	20.8	
≥ 5	14	19.2	**0.030**
Largest tumor diameter			
< 5 cm	73	34.1	
≥ 5 cm	15	37.0	0.999
CEA level (before hepatectomy)			
< 5 ng/ml	35	27.9	
≥ 5 ng/mL	52	38.5	0.438
CA19-9 level (before hepatectomy)			
< 38 U/mL	63	29.6	
≥ 38 U/mL	24	49.7	0.759
**Stage (primary tumor)**			
I–III	42	43.7	
IV	46	27.3	**0.041**
**Liver metastasis classification**			
H1	55	47.6	
H2–3	28	19.9	**0.013**
**Prognosis grade classification**			
Grade A	50	48.7	
Grade B, C	32	17.3	**0.005**
Primary tumor differentiation			
Well-differentiated	46	35.5	
Others	42	34.5	0.775
lympatic invasion (primary tumor)			
Negative	29	41.5	
Positive	59	31.6	0.161
**venous invasion (primary tumor)**			
Negative	59	43.1	
Positive	29	19.9	**0.021**
N			
Negative	27	42.3	
Positive	61	31.3	0.057
**Beppu’s** nomogram **score**			
< 6	34	49.2	
≥ 6	54	26.5	**0.027 1.994 (1.083–3.672) 0.027**
**Chemotherapy before hepatectomy**			
Absent	30	46.8	
Present	58	29.4	**0.029 1.962 (1.092–3.524) 0.024**
Operative procedure			
Laparoscopic hepatectomy	26	36.1	
Open hepatectomy	62	34.3	0.493

*BMI* body mass index, *mGPS* modified Glasgow prognostic score, *NLR* neutorophil lymphocyte ratio, *PNI* prognostic nutrition index, *CEA* carcinoembryonic antigen, *CA19-9* carbohydrate antigen 19-9

**Fig. 1:**
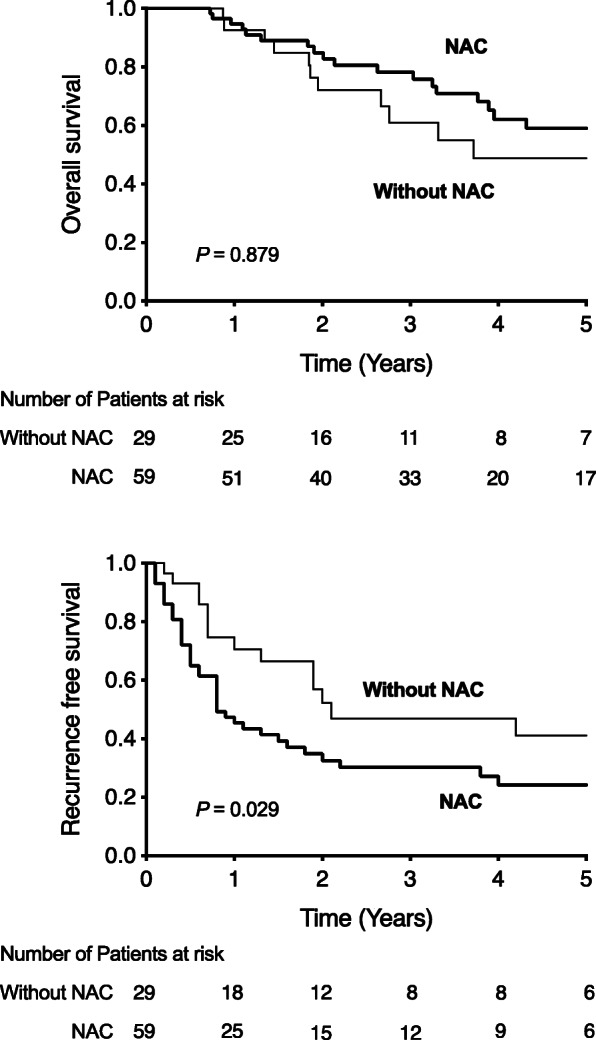
Overall survival (OS) and recurrence-free survival (RFS) after initial treatment for colorectal liver metastasis. RFS rate was significantly better in patients who did not receive neoadjuvant chemotherapy (the NAC− group) than in those who received neoadjuvant chemotherapy (the NAC+ group) (*P* = 0.029). The OS rate was not different between the two cases

Histologically, 46 (52%) primary tumors were well-differentiated adenocarcinomas; the remaining were of various types. Lymphatic invasion was negative in 29 (33%) and positive in 59 (67%) patients. Venous invasion was negative in 59 (67%) and positive in 29 (33%) patients. Regional lymph node metastasis around the primary tumor was negative in 27 (31%) patients. Beppu’s nomogram score was > 6 points in 54 (61%) and > 10 in 31 (39%) patients. NAC was administered in 58 (67%) patients: 5-fluorouracil (5FU), leucovorin (LV), and oxaliplatin (FOLFOX) or capecitabine and oxaliplatin (CAPOX) (*n* = 34); 5FU, LV, and irinotecan (FOLFIRI, *n* = 10); and tegafur/gimeracil/oteracil (TS-1) or tegafur/uracil with either LV (UFT/UZEL) or capecitabine (*n* = 14). The median interval period between NAC and hepatectomy was 48 (19–131) days. Major hepatectomy (resection of three or more Couinaud segments) was performed in 20 (23%) patients. Laparoscopic hepatectomy was performed in 26 (29.5%) patients. The Kaplan-Meier of OS and RFS was presented in Figure 1. OS was not different in both group (*P* = 0.879); however, RFS was significantly shorter in the NAC+ group than in the NAC− group (*P* = 0.029).

### Univariate and multivariate analyses for factors associated with the 3-year RFS rate of patients with resectable CRLM

On univariate analysis, the following five factors were significantly associated with a low RFS rate: tumor number ≥ 5 (*P* = 0.03), TNM stage IV at the time of surgery (*P* = 0.041), liver metastasis classification H2–3 (*P* = 0.013), prognosis grade classification B–C (*P* = 0.005), vascular invasion (*P* = 0.021), Beppu’s nomogram score ≥ 6 (*P* = 0.027), and NAC (*P* = 0.029) (Table 1). On multivariate analysis, a Beppu’s nomogram score ≥ 6 (hazard ratio, 1.994, *P* = 0.027) and NAC (hazard ratio, 1.962, *P* = 0.024) were independent risk factors for RFS (Table 1).

### Clinicopathological characteristics of the NAC+ and NAC− groups

Table 2 compares the characteristics of the patients who received NAC (NAC+ group) and those who did not (NAC− group). There were no significant differences in the general conditions of the groups at the time of hepatectomy. However, values related to the primary tumor, including the incidence of lymph node metastasis (*P* = 0.012) and vascular invasion (*P* = 0.009), were higher in the NAC+ group than in the NAC− group.

**Table 2:** Comparison of patients’ characteristics between patients with neoadjuvant chemotherapy and without neoadjuvant chemotherapy

	NAC (–) (*n* = 30)	NAC (+) (*n* = 58)	*P*-value
Male sex	21 (72%)	42 (71%)	0.904
Age (years)	73.5 (35–86)	69 (48–85)	0.026
BMI (kg/m^2^)	22 (16–34)	23 (15–30)	0.940
Location (colon)	11 (38%)	30 (51%)	0.254
Location (right side)	3 (10%)	9 (16%)	0.585
Timing of liver metastasis (synchronous)	13 (43%)	29 (50%)	0.703
Synchronous lung metastasis	0	5 (9%)	0.128
Tumor number (multiple)	14 (47%)	29 (50%)	0.938
Tumor number	1.5 (1–7)	1 (1–15)	0.521
**CEA**	**13.9 (2–1224)**	**5.25 (1.2–111.1)**	**0.011**
CA19-9	12.5 (2–642)	10.5 (2–2587.8)	0.989
Primary tumor differentiation (well)			
**N 1**	**15 (50%)**	**46 (79%)**	**0.012**
**Ly 1**	**14 (47%)**	**45 (78%)**	**0.009**
Beppu’s nomogram score 6 <	16 (53%)	38 (66%)	0.403
**Beppu’s** nomogram **score > 10**	**5 (17%)**	**26 (45%)**	**0.013**
Beppu’s nomogram score			0.092
Clavien-Dindo classification	3	11	0.372
Laparoscopic hepatectomy	12 (40%)	14 (24%)	0.088
Operation time	322 (86–596)	356 (127–727)	0.253
Intraoperative bleeding	205 (20–4000)	290 (20–3020)	0.379
PNI	46 (28–81)	46 (34–61)	0.605
NLR	2.5 (0.3–9.4)	1.9 (0.6–6.2)	0.154
GPS	6	13	0.949
mGPS	3	10	0.356

Variables in bold are statistically significant (*P* < 0.05). Continuous variables are expressed as median (range). Qualitative variables are expressed as number (%).*BMI* body mass index, *NAC* neoadjuvant chemotherapy, *NLR* neutrophil-to-lymphocyte ratio, *PNI* prognostic nutritional index, *GPS* Glasgow prognostic score, *mGPS* modified Glasgow prognostic score, *CEA* carcinoembryonic antigen, *CA19-9* carbohydrate antigen 19-9

### Prognostic impact of NAC after IPTW

**Fig. 2:**
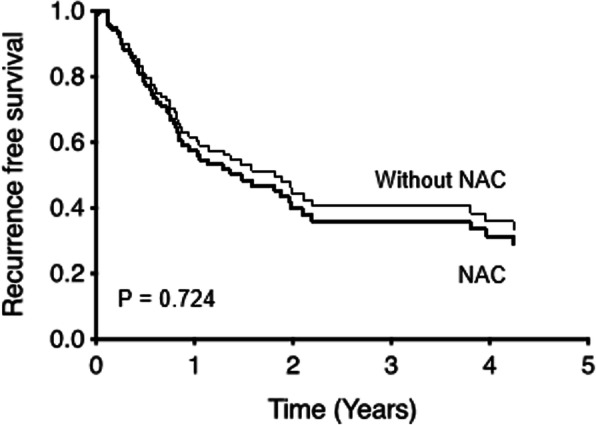
Inverse probability of treatment weighting (IPTW). IPTW shows there is no significant difference in the recurrence-free survival (RFS) rate between the patients who received neoadjuvant chemotherapy (the NAC+ group) and those who did not (the NAC− group) (*P* = 0.724)

After IPTW, there was no significant difference in the RFS rate between the NAC+ and NAC− groups (*P* = 0.724) (Table 3, Fig. 2).

**Table 3:** Unadjusted and adjusted hazard ratios for resection in patients with resectable colorectal liver metastases without neoadjuvant chemotherapy versus neoadjuvant chemotherapy

Endpoint	Crude			Adjusted^a^			IPTW^b^		
	HR	95% CI	*P*-value	HR	95% CI	*P*-value	HR	95% CI	*P*-value
RFS	1.942	1.057–3.568	0.032	1.616	0.831–3.144	0.157	1.141	0.547–2.380	0.724

^a^Adjusted for variable such as those were significant in the univariate analysis

^b^Adjusted by IPTW

*HR* hazard ratio, *IPTW* inverse probability of treatment weighing

## Discussion

This study showed the following for patients with resectable CRLM: NAC does not improve long-term prognosis, even when radical resection is performed; low Beppu’s nomogram scores are strongly associated with favorable long-term prognosis; and upfront hepatectomy is an effective treatment strategy. The aims of NAC include preservation of the remnant liver volume by tumor shrinkage and securing the surgical margin. NAC is an early treatment for micro-metastases, and determination of its efficacy is critical. Although evidence of NAC efficacy in various cancers is increasing [13, 14], there are no data supporting its use in CRLM.

In this study, lymph node metastasis, lymphatic invasion, and high Beppu’s nomogram scores were more likely in patients who received NAC than in those who did not. However, NAC did not improve prognosis, even after the background characteristics of the two groups were aligned via IPTW.

The 2016 revised ESMO guideline recommends upfront hepatectomy for patients with clearly resectable CRLM and favorable prognostic indicators [15]. However, in advanced cases with high hepatic tumor loads and multiple tumors, perioperative systemic chemotherapy is essential for down-staging unresectable CRLM. Moreover, shortening the interval between NAC and hepatectomy improves the outcome in advanced CRLM cases [16]. Along with advances in surgical technology such as two-stage hepatectomy (e.g., liver partition and portal vein ligation for staged hepatectomy), the use of NAC has increased the number of cases in which conversion surgery is possible [17].

The present study showed significant correlation between Beppu’s nomogram score and prognosis. This relatively simple nomogram was easily applied in the patients of our study. The usefulness of the nomogram, which was proposed in 2004, was thought to be unclear as the treatment policy changed as times changed. However, it is interesting that this nomogram was useful even though chemotherapy regimen has changed during the 13-year study period.

This study had a few limitations. First, it was retrospective and based on a single-center experience. Second, the relatively small sample size made it difficult to draw statistical inferences. Finally, the NAC regimens varied considerably. Considering the possibility of selection bias due to this background, propensity score matching using IPTW analysis was performed. Therefore, it is not easy to conclude whether or not NAC is efficacious for improving long-term survival in patients with CRLM. However, it is difficult to confirm our results by performing randomized controlled trials at multiple facilities due to recent increase in negative opinions about NAC for resectable CRLM [18, 19]. Beppu’s nomogram score easily calculated and precisely predicted the long-term prognosis. However, it is not the only available predictor. Several reports have identified other potential biomarkers for predicting the prognosis [20]. These include circulating tumor DNA (ctDNA) for the prediction of CRC recurrence [21] and circulating tumor cells (CTCs) [22] for the prediction of CRLM recurrence after radical resection.

In conclusion, NAC does not improve recurrence-free survival for CRLM. Upfront surgery without neoadjuvant systemic chemotherapy could be considered for resectable CRLM. Beppu’s nomogram score is a potential tool for predicting prognosis.

## Data Availability

The data that support the findings of this study are available from the corresponding author upon reasonable request.

## Abbreviations

CRC: Colorectal cancer
CRLM: Colorectal liver metastasis
NAC: Neoadjuvant chemotherapy
DFS: Disease-free survival
5FU: 5-Fluorouracil
LV: Leucovorin
CA19-9: Carbohydrate antigen 19-9
CT: Computed tomography
OS: Overall survival
RFS: Recurrence-free survival
CI: Confidence interval
IPTW: Inverse probability of treatment weighting
ctDNA: Circulating tumor DNA
CTC: Circulating tumor cell
